# A new species of *Pima* Hulst, 1888 from China (Lepidoptera, Pyralidae, Phycitinae), with a key to Holarctic species

**DOI:** 10.3897/zookeys.975.56763

**Published:** 2020-10-12

**Authors:** Linlin Yang, Yingdang Ren

**Affiliations:** 1 Institute of Plant Protection, Henan Academy of Agricultural Sciences, Henan Key Laboratory of Crop Pest Control, Key Laboratory of Integrated Pest Management on Crops in Southern Region of North China, Zhengzhou 450002, China Henan Academy of Agricultural Science Zhengzhou China

**Keywords:** COI, key, new species, *Pima boisduvaliella*, *Pima tristriata*, snout moths, taxonomy

## Abstract

*Pima tristriata***sp. nov.** is described as new to science based on specimens collected from the Ningxia Hui Autonomous Region, China, and *P. boisduvaliella* (Guenée, 1845) is also treated here for comparison. DNA barcodes of the two species are provided, together with a neighbor-joining tree for species delimitation. A key to the Holarctic species and a distribution map of the Chinese species are presented.

## Introduction

The genus *Pima* was established by Hulst (1888) with *Pima fosterella* Hulst as the type species. Ragonot (1889, 1893) referred *P. fosterella* and the other congeneric American species to *Epischnia* Hübner, 1825. Heinrich (1956) revised the genus *Pima* from America, pointing out that *Epischnia* as defined by Ragonot was a composite of several disparate species and that none of them agreed with the type species of *Epischnia*, and transferred eight species to *Pima*. Neunzig (2003) treated nine species of *Pima* from North America, described one new species and proposed two synonyms. Leraut (2014) treated five species from Europe, including one new species and two new combinations. Tsvetkov (2016) described *P. transfusor* Tsvetkov from the South Urals. Moreno and Gastón (2017) transferred four species to *Pima*. Falck et al. (2019) described *P. tricolorella* Falck, Karsholt & Slamka from the Canary Islands of Spain. Slamka (2019) reviewed the genus in Europe, synonymized *Palloria* Amsel with *Pima* and *Pima leucomixtella* (Ragonot) with *Pima christophori* (Ragonot), transferred *Epiepischnia keredjella* Amsel and *Epischnia trifidella* Zerny to *Pima*, and described three new species. Twenty-four species have hitherto been assigned to *Pima* worldwide, mainly distributed in North America and Europe.

Two species, *Pima boisduvaliella* (Guenée) and *P. trifidella* (Zerny) were reported from China before this study. We herein describe one new species, *Pima tristriata* sp. nov., provide DNA barcodes of the new species and *P. boisduvaliella* (Guenée), and a neighbor-joining tree covering seven species for species delimitation. A key to the known Holarctic species of the genus *Pima* is also provided.

## Materials and methods

The examined specimens in this study were collected by light traps in the Ningxia Hui Autonomous Region, China. Morphological terminology follows Heinrich (1956). Genitalia and wings were dissected and mounted according to the methods introduced by Li (2002). Illustrations were prepared using a Leica DM750 microscope, and refined in Photoshop CS4 software. Photographs of adults were taken with a Leica M205A stereo microscope. The cartographic illustration was made using DIVA-GIS 7.5 (Hijmans et al. 2005). All specimens examined, including the holotype of the new species, are deposited in the Insect Collection of Nankai University, Tianjin, China (**NKU**).

DNA was extracted from dry adult specimens using Qiagen DNeasy Blood & Tissue Kit, with the genitalia mounted on slides as vouchers. Samples were amplified using the primers LCO1490 and HCO2198 (Folmer et al. 1994) in 25 μl reaction volume: 0.75 μl of each primer (10 mM; Sangon Biotech), 2 μl DNA template, 12.5 μl mixture (KOD One PCR Master Mix; TOYOBO), and 9 μl ddH_2_O. PCR reaction conditions used were as follows: 35 cycles of 98 °C for 10 s, 55 °C for 5 s, 68 °C for 1 s; then a 4 °C hold. A weak electrophoretic band of the new species was obtained, and the PCR product was recovered (SanPrep Column DNA Gel Extraction Kit; Sangon Biotech) and cloned (Hieff CloneTM Zero TOPO-TA Cloning Kit; Sangon Biotech). Positive plasmids were sent to Sangon Biotech (Shanghai, China) for sequencing.

Genetic distance estimation and neighbor-joining analysis were conducted in MEGA X using the Kimura 2-Parameter model. Thirty-eight sequences were used in the analyses: one new sequence from a paratype of *P. tristriata* sp. nov. (GenBank accession number MT749678) and three new ones from Chinese specimens of *P. boisduvaliella* (GenBank accession numbers MT734539, MT734540, MT734541), the others from BOLD (Ratnasingham and Hebert 2007). The extreme values of the interspecific and intraspecific distances were presented in Table 2, and the NJ tree was shown in Fig. 8.

## Taxonomy

### Pima

Taxon classificationAnimaliaLepidoptera Pyralidae

Hulst, 1888

4F39C776-8259-5C70-9172-E35C60509EA8

Pima Hulst, 1888: 114. Type species: Pima fosterella Hulst, 1888, by original designation and monotypy
Palloria Amsel, 1961: 362. Type species: Palloria bicornutella Amsel, 1961

**Figures 1, 2.**
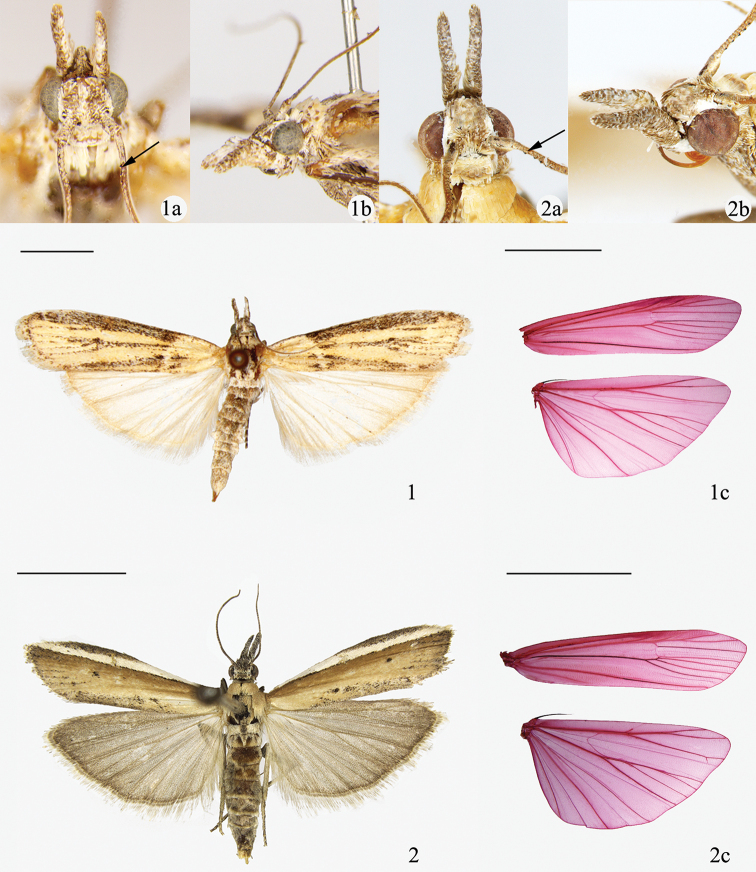
Adult *Pima* species. **1***P. tristriata* sp. nov., holotype, male **1a** dorsal view of head, holotype, male **1b** lateral view of head, holotype, male **1c** wing venation, paratype, female, DYL01090 **2***P. boisduvaliella*, female **2a** dorsal view of head, male **2b** lateral view of head, male **2c** wing venation, female, WYQ13200. Scale bars: 5.0 mm.

**Figures 3–6.**
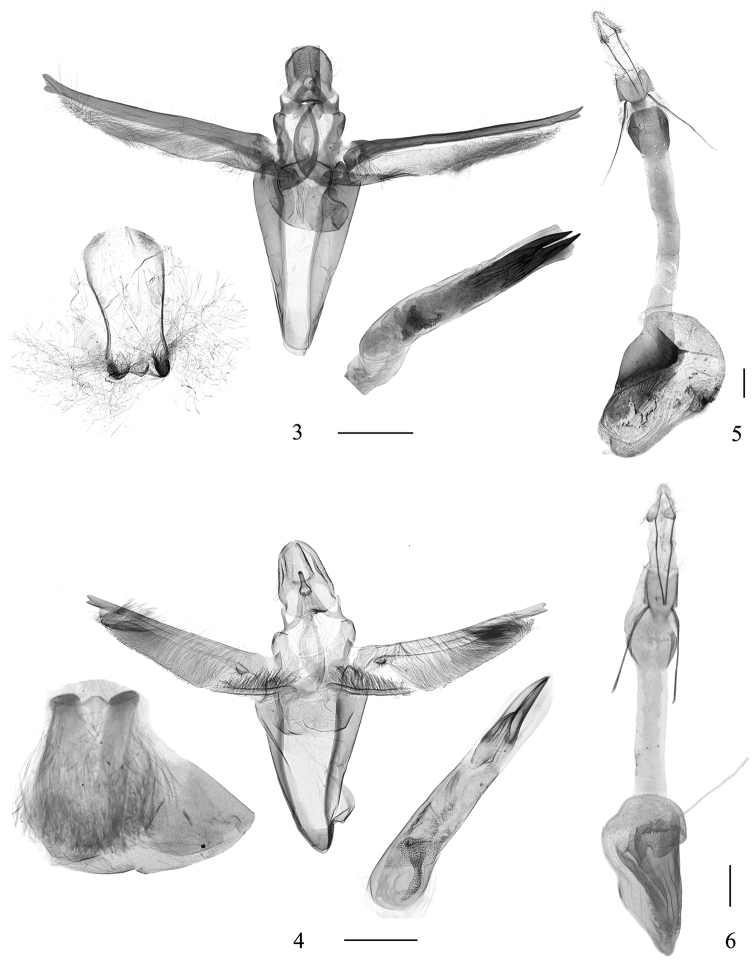
Genitalia of *Pima* species. **3, 4** Male genitalia **3***P. tristriata* sp. nov., paratype, YLL18044 **4***P. boisduvaliella*, DYL01090 **5, 6** Female genitalia **5***P. tristriata* sp. nov., paratype, LJY10110 **6***P. boisduvaliella*, DYL00331. Scale bars: 0.5 mm.

**Diagnostic characters.***Pima* is characterized by the male basal few flagellomeres shallowly incurved and containing a row of minute, tooth-like spines (Figs 1a, 2a), third segment of the labial palpus projected forward (Figs 1b, 2b); the forewing usually having a white subcostal streak (absent in *P. keredjella*, *P. milka*, *P. parkerella*, *P. pempeliella*, *P. transfusor* and *P. tristriata* sp. nov.), with 11 veins (Figs 1c, 2c), R_2_ approximate to the stalk of R_3+4_ + R_5_, R_3+4_ stalked with R_5_ of less than half their lengths, M_2_, M_3_ and CuA_1_ free; the hindwing with 10 veins (Figs 1c, 2c), Rs and M_1_ shortly stalked, M_2_ and M_3_ stalked for over half their length, CuA_1_ and M_2_+ M_3_ shortly stalked; apical process of gnathos short and stout, transtilla absent, the broad costa of the narrowed valva with a blunt, slightly forked apex (more pointed and not forked in *P. christophori*, *P. leucoloma*, *P. pempeliella*, and *P. trifidella*), the uncus with a broad base and a short pair of lateral lobes, the aedeagus with two stout cornuti (one cornutus in *P. trifidella*) in male genitalia (Figs 3, 4); the ductus bursae ribbon-like, the stout corpus bursae scobinate-granulate and usually with sclerotized patches or folds in female genitalia (Figs 5, 6).

*Pima* resembles *Epischnia* Hübner, but they can be separated by the following characters: in *Pima*, the male flagellum with a row of tooth-like spines near the base, the labial palpus with terminal two segments approximately of equal length; male genitalia with a broad, apically slightly forked costa, and two stout cornuti in the aedeagus; female genitalia with a strongly sclerotized, funnel-shaped antrum, the corpus bursae scobinate-granulate throughout and with sclerotized patches or folds. Whereas, in *Epischnia*, the male flagellum lacks a tooth-like spine, the third of the labial palpus is less than half the length of the second; the costa is weak and not forked at the apex, and the aedeagus has a bunch of spinules in the male genitalia; the antrum is weak or represented by a band-shaped plate, the corpus bursae is smooth on the inner surface except for one big sclerotized plate or a line of small thorns and one bunch of spinules in the female genitalia.

### Key to Holarctic species of the genus *Pima*

**Table:** 

1	Forewing with distinct white subcostal streak	**2**
–	Forewing with obscure white subcostal streak or absent	**17**
2	Forewing ground color creamy-whitish, with a distinct longitudinal brown streak under white subcostal streak (Amsel 1961: pl. 3, fig. 181; Slamka 2019: pl. 22, fig. 152)	***P. keredjella***
–	Forewing ground color yellowish, grayish or brown, without distinct streak under white subcostal streak	**3**
3	White subcostal streak conspicuously developed only form base of costa to the antemedial line (Falck, Karsholt and Slamka 2019: figs 5, 6)	***P. tricolorella***
–	White subcostal streak well developed along whole length of forewing	**4**
4	Costa of valva has a more pointed and not forked apex	**5**
–	Costa of valva has a blunt, slightly forked apex	**7**
5	Aedoeagus has a single cornutus (Slamka 2019: pl. 78, fig. 156)	***P. trifidella***
–	Aedoeagus has two cornuti	**6**
6	Forewing ground color grayish, with faint postmedial line (Vives Moreno and Gastón 2017: fig. 19; Slamka 2019: pl. 22, figs 154a–e)	***P. leucoloma***
–	Forewing ground color pale yellow, without transverse line (Leraut 2014: pl. 41 fig. 8; Slamka 2019: pl. 22, figs 155a–d)	***P. christophori***
7	Corpus bursae with a slug-shaped sclerotization	**8**
–	Corpus bursae without the above sclerotization	**13**
8	Two cornuti ca equal thickness	**9**
–	Shorter cornutus broader than the longer one	**10**
9	Corpus bursae heart-shaped (Fig. 6)	***P. boisduvaliella***
–	Corpus bursae oblong (Heinrich 1956: fig. 777)	***P. albiplagiatella***
10	Shorter cornutus significantly shorter than the longer one (Leraut 2014: text fig. 121c; Slamka 2019: pl. 73, fig. 146a, pl. 74, fig. 146b–d); Corpus bursae ca 1.5× length of its medial width (Leraut 2014: fig. 122a; Slamka 2019: pl. 154, fig. 146)	***P. tabulella***
–	Shorter cornutus slightly shorter than the longer one; Corpus bursae more than double length of its medial width	**11**
11	Shorter cornutus broad at base, abruptly tapered to apex (Slamka 2019: pl. 74, figs 148)	***P. marocana***
–	Shorter cornutus gradually tapered to apex	**12**
12	Gnathos-arms stouter (Slamka 2019: pl. 74, fig. 147); Corpus bursae sclerotized in posterior three-quarters (Leraut 2014: text fig. 122c; Slamka 2019: pl. 155, figs 147a, b)	***P. aureliae***
–	Gnathos-arms narrower (Slamka 2019: pl. 75, fig. 149); Corpus bursae sclerotized in posterior three-quarters (Slamka 2019: pl. 155, fig. 149a, pl. 156, fig. 149b–c)	***P. yllai***
13	Corpus bursae oblong	**14**
–	Corpus bursae nearly rounded	**15**
14	Corpus bursae without hump-shaped protuberance (Neunzig 2003: text fig. 3)	***P. occidentalis***
–	Corpus bursae with a sclerotized hump (Slamka 2019: pl. 156, figs 150a, b)	***P. vilhelmseni***
15	Forewing pale (Neunzig 2003: pl. 1, fig. 1); corpus bursae not sclerotized anteriorly (Heinrich 1956: fig. 783; Neunzig 2003: fig. 2c)	***P. fosterella***
–	Forewing darker; corpus bursae sclerotized anteriorly	**16**
16	Forewing salmon pink below white subcostal streak (Neunzig 2003: pl. 1, fig. 6); antrum subovate (Heinrich 1956: fig. 780)	***P. fulvirugella***
–	Forewing dark gray to blackish brown below white subcostal streak (Neunzig 2003: pl. 1, fig. 7; Leraut 2014: pl. 41, fig. 11); antrum funnel-shaped (Heinrich 1956: figs 778, 779)	***P. albocostalialis***
17	Forewing with faint antemedial and postmedial lines	**18**
–	Forewing without transverse lines	**22**
18	Costa of valva not forked at apex	**19**
–	Costa of valva slightly forked at apex	**20**
19	Juxta V-shaped, aedeagus significantly shorter than the valva, clasper present (Roesler 1990: fig. 9); apophyses posteriores slightly shorter than anteriores (Roesler 1990: fig. 10)	***P. milka***
–	Juxta U-shaped, aedeagus as long as the valva, clasper absent (Roesler 1990: fig. 11; Slamka 2019: pl. 76, fig. 153); apophyses posteriores significantly shorter than anteriores (Roesler 1990: fig. 12; Slamka 2019: pl. 157, fig. 153)	***P. pempeliella***
20	Forewing with longitudinal grayish black streaks along costa and dorsum	**21**
–	Forewing without longitudinal fuscous streaks (Tsvetkov 2016: fig. 1)	***P. transfusor***
21	Forewing with a longitudinal grayish black streaks along lower margin of cell (Fig. 1); costa projected beyond apex of valva (Fig. 3); corpus bursae heart-shaped (Fig. 5)	***P. tristriata* sp. nov.**
–	Forewing without longitudinal streaks along lower margin of cell (Neunzig 2003: pl. 1, fig. 10); costa not projected beyond apex of valva (Heinrich 1956: fig. 306); corpus bursae more rounded, with a projecting shield at junction with ductus bursae (Heinrich 1956: fig. 782)	***P. parkerella***
22	Forewing more nearly uniform, without contrasting longitudinal lines (Neunzig 2003: pl. 1, figs 11, 12)	***P. fergusoni***
–	Forewing more black along veins (Neunzig 2003: pl. 1, figs 8, 9)	***P. granitella***

### Pima tristriata sp. nov.

Taxon classificationAnimaliaLepidoptera Pyralidae

BE40F1B0-4B54-5E66-824B-829DC2AE57FE

http://zoobank.org/06B4BDCD-1419-4541-9BAA-D42E2FAA01DC

Figures 1 , 3 , 5

#### Type material.

***Holotype***: China: • ♂; Shapotou (37°31'N, 105°10'E), Zhongwei, Ningxia Hui Autonomous Region; alt. 1140 m; [?]-v-1985; Guo-Dong Ren leg. ***Paratypes***: China: • 7♂; same data as the holotype; genitalia nos. DYL01079, DYL01080, RYD04466 • 3♂, 2♀; same data as the holotype except dated 23-iv-1987; genitalia nos. YLL18042♂, YLL18044♂ • 2 ♀; Gantang (37°27'N, 105°32'E), Zhongwei, Ningxia; 23-v-1987; Guo-Dong Ren leg.; genitalia no. DYL01090.

#### Diagnosis.

The new species can be easily distinguished from its congeners in having one longitudinal grayish black streak along the costa, dorsum, and lower margin of cell respectively, whereas, most of the other congeners have a white subcostal streak. It is superficially similar to *P. parkerella* (Schaus), but with differences in genitalia: juxta with globular lateral lobes, costa projected beyond apex of valva, and corpus bursae heart-shaped in the new species; juxta with short finger-like lateral lobes, costa terminated at end of valva, and corpus bursae rounded in *P. parkerella*. It resembles *Pima boisduvaliella* (Guenée) in genitalia except for some slight differences: lateral lobes the juxta is globular, the vinculum is ca 2× length of its greatest width, the aedeagus is approximately equal to valva in length in the male genitalia, and the corpus bursae has an irregular sclerotized plate in the female genitalia. In *P. boisduvaliella*, lateral lobes the juxta is slender, finger-like, the vinculum is ca 1.5× length of its greatest width, and the aedeagus is 1.2× length of valva in the male genitalia; the corpus bursae has a couple of tortuous, sclerotized plates in the female genitalia.

#### Description.

***Adult*** (Fig. 1). Wingspan 25.5–31.0 mm. Head (Fig. 1a, b) grayish white. Antenna grayish white, scape ca 1.5× as long as wide, flagellum of male with short cilia, of female pubescent. Labial palpus of male grayish white mixed with a few brown scales, of female brown mixed with a few grayish white scales; first and second segments obliquely upturned, third second projected forward; third segment as long as second, twice as long as first. Maxillary palpus minute, grayish brown, in form of an aigrette. Patagium, tegula and thorax pale yellow, mottled a few brown scales. Forewing yellow, costa dorsum and lower margin of cell overlaid with a longitudinal grayish black streak respectively, more or less peppering of whitish scales; some scattered black dotting along veins and termen; antemedial line white, arched, white, from costal 1/5 slightly oblique to dorsum 1/4, inner bordering ashy black on lower half, outer edging of grayish brown; postmedial line indistinct; discal spots brown, separated; postmedial line black, obscure; cilia yellowish write. Hindwing pale gray, cilia grayish white.

***Male genitalia*** (Fig. 3). Uncus oval, lateral margins enfolded at distal half. Apical process of gnathos conical, ca 1/3 length of uncus. Transtilla absent. Valva narrow, 5× as long as wide; clasper a narrowed triangular process, with a globular, haired base; costa stout, slightly longer than and ca 2/3 width of valva, its apex blunt, slightly forked; sacculus ca 2/5 length of valva, broader at base, tapering toward pointed apex, bearing dense, spine-like hairs along ventral margin. Juxta a broad, semicircular plate, with a pair of short, globular lateral lobes. Vinculum twice as long as its greatest width, narrowly rounded anteriorly. Aedeagus nearly as long as valva, slightly curved towards base, with a tuft of granulations near base; Cornuti two stout thorns, longer one slightly less than half length of aedeagus. Culcita one pair of long hair tufts, 2/3 length of valva.

***Female genitalia*** (Fig. 5). Ovipositor triangular, 3× as long as wide. Apophyses posteriores slender, 3/4 length of apophyses anteriores. Eighth tergite 2/3 length of its width. Antrum strongly sclerotized, funnel-shaped, broader than eighth segment. Ductus bursae sclerotized, 1.2× as long as corpus bursae, of nearly equal width throughout, slightly broader at junction with corpus bursae. Corpus bursae heart-shaped, scobinate-granulate on inner surface, with two sclerotized patches: one oval sclerotized plate near middle; one irregular large plate from junction with ductus bursae to anterior 1/3, its posterior half smooth, forming a shallow fold along its edge, anterior half granulated and wrinkled. Ductus seminalis from posterior margin of corpus bursae.

#### DNA barcode.

One DNA barcode from a female paratype was obtained and deposited in GenBank (accession numbers: MT749678), DNA voucher slide no. DNAYLL18119.

#### Etymology.

The specific name is derived from the Latin prefix *tri*-, meaning three, and the Latin word *striatus*, meaning streak, referring to three grayish black streaks on the forewing.

#### Distribution.

China (Ningxia).

#### Host plant.

Unknown.

### Pima boisduvaliella

Taxon classificationAnimaliaLepidoptera Pyralidae

(Guenée, 1845)

2E9FF304-6CDD-5597-8611-39DD11389407

Figures 2 , 4 , 6

Epischnia boisduvaliella Guenée, 1845: 319.
Anerastia farrella Curtis, 1850: 114.
Myelois lafauryella Constant, 1865: 189.
Pima boisduvaliella (Guenée): Hannemann 1964: 180.

#### Diagnosis.

Adults (Fig. 2) with wingspan 15.0–22.0 mm. *Pima boisduvaliella* is characterized by the yellowish brown forewing with a white subcostal streak; the elongate valva with a well-developed costa that produced and weakly notched apically, the broad semicircular juxta with a pair of short, finger-like lateral lobes, the V-shaped vinculum ca 1.5× length of its greatest width, and the aedeagus with two thorns that slightly less than half the length of the aedeagus in the male genitalia (Fig. 4); the rounded antrum, the heart-shaped corpus bursae with dense microtrichia in anterior 1/3, with a small oval sclerotized plate and a couple of tortuous, sclerotized plates in the female genitalia (Fig. 6).

Three DNA barcodes were obtained and deposited in GenBank: a male collected on August 19, 2007 at alt. 2178 m in Mt. Xinglong, Yuzhong County, Gansu Province, accession no. MT734539, DNA voucher slide no. DNAYLL18043; a male collected on July 24, 2013 at alt. 1461 m in Habahu, Yanchi County, Ningxia Hui Autonomous Region, accession no. MT734540, DNA voucher slide no. DNAYLL18076; a male collected on August 3, 2010 at alt. 1836 m in Shuimogou, Mt. Helan, Alxa Zuoqi, Inner Mongolia Autonomous Region, accession no. MT734541, DNA voucher slide no. DNAYLL18118.

#### Distribution.

**Figure 7.**
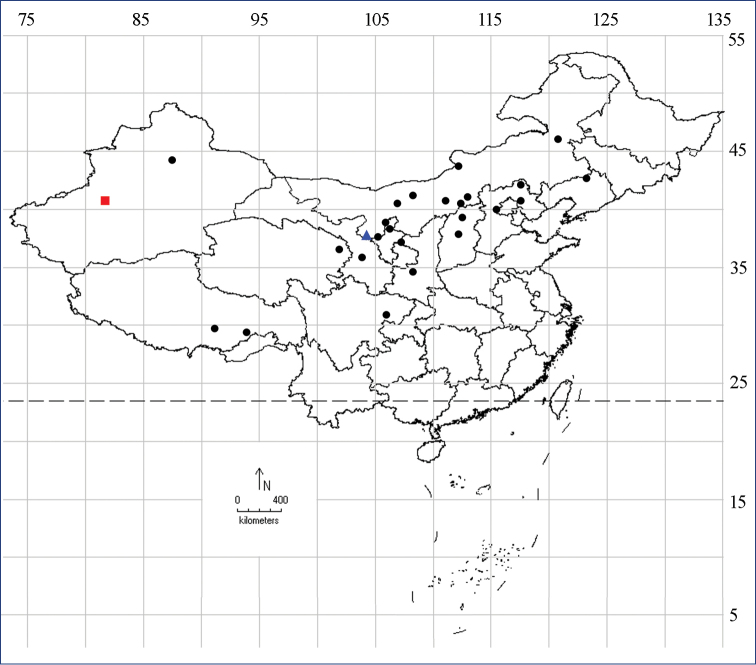
Geographical distribution of *Pima* in China: *P. tristriata* sp. nov. (triangle), *P. boisduvaliella* (circle); *P. trifidella* (square).

China (Gansu, Hebei, Inner Mongolia, Liaoning, Ningxia, Qinghai, Shaanxi, Shanxi, Xinjiang, Xizang) (Fig. 7), Europe (Slamka 2019: 128, fig. 145), Canada, USA.

#### Host plants.

Leguminosae: *Anthyllis vulneraria* L., *Astragalus dasyanthus* Pall., *Astracantha arnacanthoides*, *Lathyrus japonicus* Willd., *Lotus corniculatus* L., *Ononis spinosa* L., *O. arvensis* L., *Hibiscus esculentus* (Heinrich 1956; Leraut 2014; Slamka 2019).

## Discussion

*Pima* is a genus containing 25 species of which 15 are Palaearctic (Roesler 1990; Leraut 2014; Tsvetkov 2016; Vives Moreno and Gastón 2017; Slamka 2019), nine species are Nearctic (Heinrich 1956; Neunzig 2003), and two are Afrotropical (Joannis 1927) (Table 1). Species of *Pima* might be expected to occur at higher elevations, as most of them were recorded from mountainous areas. In China (Fig. 7), *P. boisduvaliella* is mainly distributed in the north, but also occurred in the west, such as Xinjiang and Tibet; *P. trifidella* is distributed in Xinjiang; and *P. tristriata* sp. nov. is only found in Zhongwei, Ningxia. Adults of the two species were collected from mountain areas with altitudes ranging from 900 m to 3050 m.

**Table 1.** Distribution of the *Pima* species in the worldwide .

Species	Distribution
*P. albiplagiatella*	southeastern Canada and northeastern USA
*P. albocostalialis*	southwestern Canada, Pacific Coast states and Rocky Mountain states of USA
*P. aureliae*	Tunisia
*P. boisduvaliella*	from Europe to Central Asia, Southern Canada and Northern USA
*P. christophori*	Armenia, Georgia, Iran, Turkey, Turkmenistan
*P. difficilis*	Mozambique
*P. fergusoni*	Oregon and California of USA
*P. flavidorsella*	Mozambique
*P. fosterella*	western Canada and USA
*P. fulvirugella*	south central and southwestern Canada and Northern California
*P. granitella*	Rocky Mountain and Pacific Coast states of USA
*P. keredjella*	Iran
*P. leucoloma*	Crimea, Croatia, Cyprus, Greece, Italy, Lebanon, Spain, Syria, W Turkey, Tunisia
*P. marocana*	Morocco
*P. milka*	Iran
*P. occidentalis*	Rocky Mountain and Pacific Coast states of USA
*P. parkerella*	Montana of Canada
*P. pempeliella*	Morocco
*P. tabulella*	Altai Republic, NW Mongolia, Turkmenistan
*P. transfusor*	South Urals
*P. tricolorella*	Spain
*P. trifidella*	China
*P. tristriata* sp. nov.	China
*P. vilhelmseni*	Libya, Morocco, Tunisia
*P. yallai*	Morocco, Tunisia

**Figure 8.**
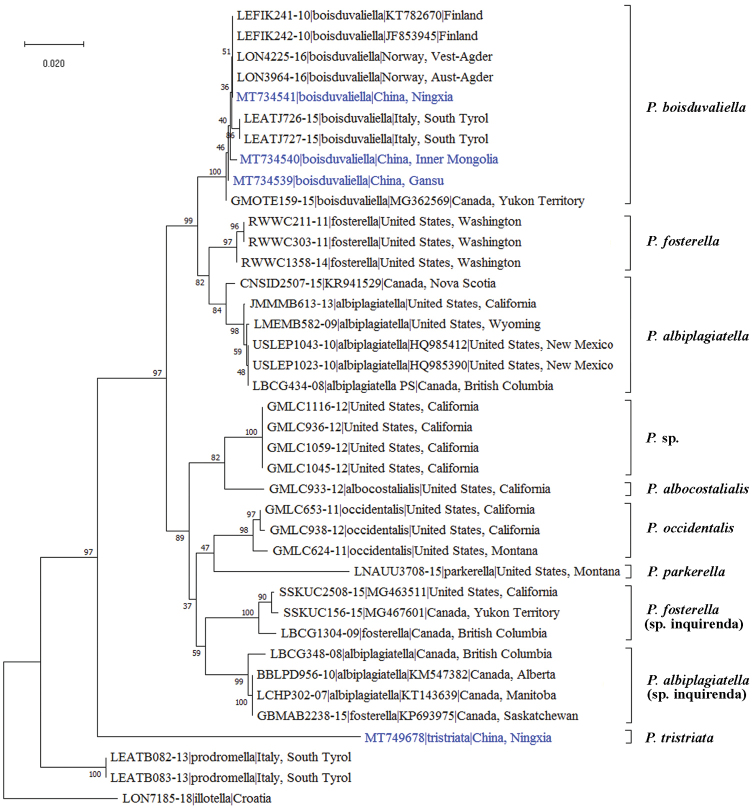
Neighbor-joining tree deduced from the cytochrome c oxidase subunit I (COI) gene sequences using MEGA X. Sequences were corrected with the Kimura two-parameter substitution model. Codon positions included were 1^st^ + 2^nd^ + 3^rd^ + non-coding. Values represented at the nodes of branches are bootstrap values (1000 replicates).

The genetic distance analysis was made based on the pairwise analysis of 38 sequences. According to the NJ bootstrap consensus tree (Fig. 8), ten well-supported clusters of *Pima* were revealed: *P. tristriata* sp. nov. is clearly distinguished from its congeners, and this is highly consistent with the morphological analysis; three specimens (LBCG348-08, BBLPD956-10, LCHP302-07) labeled *P. albiplagiatella*, two specimens (GBMAB2238-15, LBCG1304-09) labeled *P. fosterella*, and an additional two unidentified specimens (SSKUC2508-15, SSKUC156-15) might represent two unnamed species, as members show higher divergences with *P. albiplagiatella* and *P. fosterella*, here treated as *P. albiplagiatella* sp. inquirenda and *P. fosterella* sp. inquirenda. Sequence divergences among individuals (Table 2) indicated that minimal interspecific distances range from 1.7 to 2.2%, and the maximal intraspecific distances range from 0 to 1.2%. The present analysis is limited by the relatively small number of species that have been sequenced, and further study is necessary to determine the boundaries of intraspecific and interspecific distances, and whether the minor morphological difference is intraspecific variation or interspecific difference.

**Table 2.** Percentage of divergence in the cytochrome c oxidase subunit I (COI) gene sequences of the *Pima* species.

		1	2	3	4	5	6	7	8	9	10	11
**1**	*Epischnia illotella*											
**2**	*E. prodromella*	7.4										
**3**	*Pima albiplagiatella*	12.8–13	9.4–9.9	**0–1.2**								
**4**	*P. albiplagiatella* (sp. inquirenda)	12.1–12.5	9.2–9.9	5.4–6.2	**0–0.8**							
**5**	*P. fosterella*	11.1–12.5	9.4	1.7–2.2	5.2–6.4	**0**						
**6**	*P. fosterella* (sp. inquirenda)	11.4–12.3	9.0–9.2	6.2–7.5	3.5–4.7	5.4–6.0	**0.3–1.2**					
**7**	*P. albocostalialis*	12.9	9.4	5.0–5.9	5.2–5.7	5.4–5.6	6.0–6.4					
**8**	*Pima* sp.	11.3	9.4	5.2–5.9	4.7–5.5	5.4–5.7	5.9–6.0	2.6	**0**			
**9**	*P. boisduvaliella*	11.6–12.3	8.4–9.2	2.3–3.2	4.5–5.5	2.0–3.1	5.7–6.1	5.6–6.2	5.4–5.9	**0–0.6**		
**10**	*P. occidentalis*	11.7–12.1	8.5–8.7	5.4–6.4	3.9–4.9	5.5–5.8	4.6–5.2	4.7–5.0	5.9	5–5.9	**0.2–0.9**	
**11**	*P. parkerella*	14.4	12	8.1–9.1	7.6–7.8	8	7.3–8.2	6.5	7.3	8.1–9.2	6.2–6.4	
**12**	*P. tristriata* sp. nov.	15.6	13.6	13.3–13.8	15.5–15.9	13.3–13.6	15.9–16.4	15.1	15.1	12.5–13.4	15.3–15.5	18.6

Genetic distances (%) were corrected with the Kimura two-parameter (K2P) substitution model using MEGA X; extreme values of intraspecific and interspecific distances are given (the numbers in bold are the intraspecific distances).

## Supplementary Material

XML Treatment for Pima

XML Treatment for Pima tristriata

XML Treatment for Pima boisduvaliella
